# Physicochemical properties and structural dynamics of organic–inorganic hybrid [NH_3_(CH_2_)_3_NH_3_]Zn*X*_4_ (*X* = Cl and Br) crystals

**DOI:** 10.1038/s41598-021-87940-2

**Published:** 2021-04-16

**Authors:** Ae Ran Lim, Sun Ha Kim, Yong Lak Joo

**Affiliations:** 1 Department of Carbon Convergence Engineering, Jeonju University, Jeonju, 55069 Korea; 2 Department of Science Education, Jeonju University, Jeonju, 55069 Korea; 3 Korea Basic Science Institute, Seoul Western Center, Seoul, 03759 Korea; 4 Department of Chemistry, Kyungpook National University, Daegu, 41566 Korea; 5 Robert Fredrick Smith School of Chemical and Biomolecular Engineering, Cornell University, Ithaca, NY 14853 USA

**Keywords:** Materials science, Physics

## Abstract

The physical properties of the organic–inorganic hybrid crystals having the formula [NH_3_(CH_2_)_3_NH_3_]ZnX_4_ (X = Cl, Br) were investigated. The phase transition temperatures (T_C_; 268K for Cl and 272K for Br) of the two crystals bearing different halogen atoms in their skeletons were determined through differential scanning calorimetry. The thermodynamic properties of the two crystals were investigated through thermogravimetric analysis. The structural dynamics, particularly the role of the [NH_3_(CH_2_)_3_NH_3_] cation, were probed through ^1^H and ^13^C magic-angle spinning nuclear magnetic resonance spectroscopy as a function of temperature. The ^1^H and ^13^C NMR chemical shifts did not show any changes near T_C_. In addition, the ^1^H spin–lattice relaxation time (T_1ρ_) varied with temperature, whereas the ^13^C T_1ρ_ values remained nearly constant at different temperatures. The T_1ρ_ values of the atoms in [NH_3_(CH_2_)_3_NH_3_]ZnCl_4_ were higher than those in [NH_3_(CH_2_)_3_NH_3_]ZnBr_4_. The observed differences in the structural dynamics obtained from the chemical shifts and T_1ρ_ values of the two compounds can be attributed to the differences in the bond lengths and halogen atoms. These findings can provide important insights or potential applications of these crystals.

## Introduction

Organic–inorganic compounds based on hybrid perovskites, particularly [(C_*n*_H_2*n*+1_NH_3_)]_2_*BX*_4_ (*n* = 1, 2, 3,···; *B* = Mn, Co, Cu, Zn, Cd; *X* = Cl, Br) and [NH_3_(CH_2_)_*n*_NH_3_]*BX*_4_ (*n* = 2, 3,…) have attracted considerable attention in recent years. Monoammonium series [(C_*n*_H_2*n*+1_NH_3_)]_2_*BX*_4_^1–5^ and diammonium series [NH_3_(CH_2_)_*n*_NH_3_]*BX*_4_ have been extensively studied because of their relative stability and potential applications^6–11^. The physical and chemical properties of the organic–inorganic hybrid perovskites depend on the characteristics of the organic cations, geometry of the inorganic anions (metal halide ions; (*BX*_6_)^2−^ or (*BX*_4_)^2−^), and reaction stoichiometry^1–3,12–18^. For *B* = Mn, Cu, or Cd, the structure is consists of the corner shared octahedral (*BX*_6_)^2−^ alternated with organic layers. While for *B* = Co or Zn, the structures are tetrahedral (*BX*_4_)^2−^ sandwiched between layers of organic cations. These compounds have gained research attention because of the multiplicity of their crystal structures, which is correlated to the structural dynamics of the cations and anions. Organic–inorganic hybrid materials based on perovskite structures are of interest due to their potential applications^19–21^. Recently, solid-state NMR on hybrid perovskite materials has also garnered considerable attention^22–25^.

**Figure 1:**
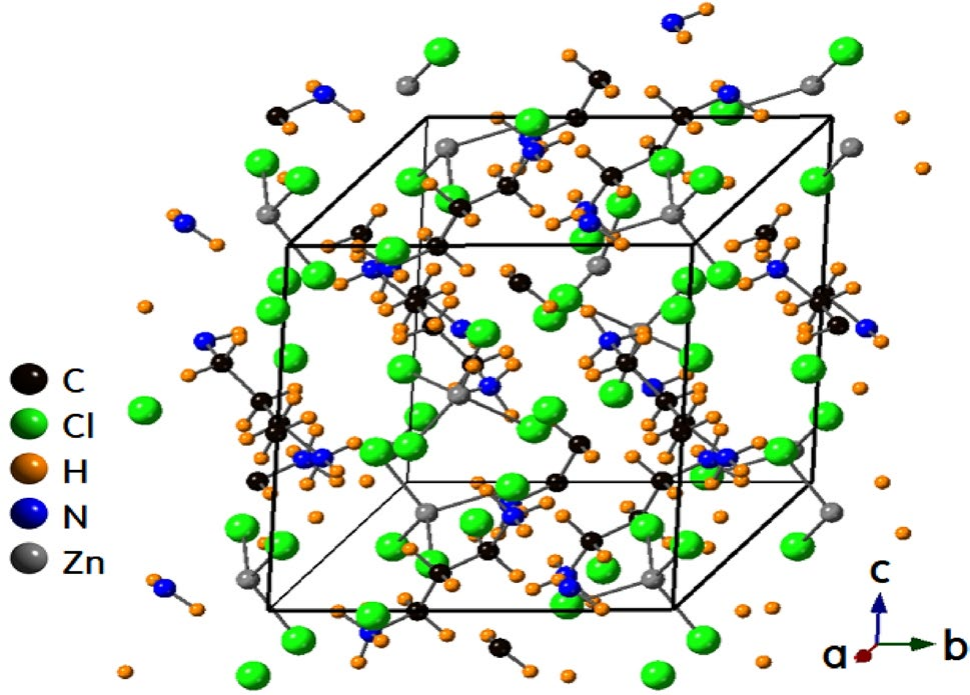
Crystal structure of [NH_3_(CH_2_)_3_NH_3_]ZnCl_4_ at room temperature.

The [NH_3_(CH_2_)_3_NH_3_]ZnCl_4_ (1,3-propanediammonium tetrachlorozincate (II)) complex (*n* = 3; *B* = Zn; *X* = Cl), a member of the diammonium [NH_3_(CH_2_)_*n*_NH_3_]*BX*_4_ series, belongs to the monoclinic crystal system (space group: P2_1_/n) at room temperature. Its lattice constants have been reported as *a* = 10.692 Å, *b* = 10.611 Å, *c* = 10.786 Å, β = 118.47°, and Z = 4^26^. Figure 1 shows the structure of the [NH_3_(CH_2_)_3_NH_3_]ZnCl_4_ crystal at 300 K (CCDC number : 1227730). The crystal structure of the [NH_3_(CH_2_)_3_NH_3_]ZnBr_4_ (1,3-propanediammonium tetrabromozincate (II)) complex (*n* = 3, *B* = Zn; *X* = Br) is identical to that of the [NH_3_(CH_2_)_3_NH_3_]ZnCl_4_ complex; both belong to the same space group. Its unit cell dimensions were determined as *a* = 11.084 Å, *b* = 10.968 Å, *c* = 11.185 Å, β = 117.07°, and Z = 4^27^. The [ZnBr_4_]^2−^ anion forms a tetrahedron interconnected via the N‒H···Br hydrogen bonds to the [NH_3_(CH_2_)_3_NH_3_] cation. There is no center of symmetry within the [NH_3_(CH_2_)_3_NH_3_] cation in [NH_3_(CH_2_)_3_NH_3_]ZnBr_4_.

To date, the structures of [NH_3_(CH_2_)_3_NH_3_]Zn*X*_4_ (*X* = Cl and Br) have been reported by Kallel et al.^22^ and Ishihara et al.^23^, who studied the single crystals of the complexes through X-ray diffraction analysis. Although [NH_3_(CH_2_)_3_NH_3_]Zn*X*_4_ (*X* = Cl and Br) has many applications, the physical properties and structural dynamics of its crystals have not been elucidated.

Herein, the phase transition temperatures and thermodynamic properties of the crystals of [NH_3_(CH_2_)_3_NH_3_]ZnCl_4_ and [NH_3_(CH_2_)_3_NH_3_]ZnBr_4_ complexes are determined by differential scanning calorimetry (DSC) and thermogravimetric analysis (TGA). The structural dynamics of the crystals of [NH_3_(CH_2_)_3_NH_3_]Zn*X*_4_ (particularly the role of the [NH_3_(CH_2_)_3_NH_3_] cation) were probed by ^1^H magic-angle spinning nuclear magnetic resonance (MAS NMR) and ^13^C MAS NMR spectroscopy as a function of temperature. The ^1^H and ^13^C NMR spectral profiles were recorded to determine the changes in the chemical shifts. A change in the chemical shift values reflects a change in the structural environment. In addition, the spin–lattice relaxation times (T_1ρ_) in the rotating frame were discussed according to the change of temperature. Based on the MAS NMR results, the effects of different halogen atoms on the hydrogen bond and carbon atoms in the [NH_3_(CH_2_)_3_NH_3_]ZnCl_4_ and [NH_3_(CH_2_)_3_NH_3_]ZnBr_4_ crystals were investgated. Moreover, comparison of the physical properties of the two crystals revealed important information regarding the basic mechanisms.

## Experimental

An aqueous solution containing NH_2_(CH_2_)_3_NH_2_·2HCl and ZnCl_2_ was slowly evaporated at 300 K to produce the single crystals of [NH_3_(CH_2_)_3_NH_3_]ZnCl_4_. Similarly, the slow evaporation at 300 K of an aqueous solution containing NH_2_(CH_2_)_3_NH_2_·2HBr and ZnBr_2_, produced the single crystals of [NH_3_(CH_2_)_3_NH_3_]ZnBr_4_. Transparent hexagonal crystals of [NH_3_(CH_2_)_3_NH_3_]ZnCl_4_ and thin-plate-shaped crystals of [NH_3_(CH_2_)_3_NH_3_]ZnBr_4_ were produced.

The structures of the [NH_3_(CH_2_)_3_NH_3_]Zn*X*_4_ (*X* = Cl and Br) crystals at 298 K were analysed using an X-ray diffraction system equipped with a Cu-Kα radiation source. And, the lattice parameters were determined by single-crystal X-ray diffraction methods at the Western Seoul Center of Korea Basic Science Institute. The crystals were mounted on a Bruker D8 Venture equipped with IμS micro-focus sealed tube Mo-Kα and a PHOTON III M14 detector.

DSC (TA, DSC 25) experiments were carried out at a scanning speed of 10 K/min in the temperature range of 190–620 K under an atmosphere of nitrogen. TGA experiments were performed on a thermogravimetric analyzer (TA Instrument) in the temperature range of 300–870 K. The same heating rate was maintained. The amounts of [NH_3_(CH_2_)_3_NH_3_]ZnCl_4_ used for the DSC and TGA experiments were 6.04 and 6.72 mg, respectively and the amounts of [NH_3_(CH_2_)_3_NH_3_]ZnBr_4_ used were 6.40 and 8.96 mg, respectively.

The NMR spectra of the crystals of [NH_3_(CH_2_)_3_NH_3_]ZnCl_4_ and [NH_3_(CH_2_)_3_NH_3_]ZnBr_4_ were recorded on a 400 MHz Avance II + Bruker solid-state NMR spectrometer equipped with 4 mm MAS probes (at KBSI, Western Seoul Center). The MAS ^1^H and ^13^C NMR experiments were conducted at the Larmor frequency of 400.13 and 100.61 MHz, respectively. It was observed that an MAS rate of 10 kHz can minimize the spinning sidebands. Tetramethylsilane (TMS) was used as the standard to record the NMR spectra. As preparation for the MAS NMR experiments, the single crystal was ground into powder. The T_1ρ_ values were obtained using a variable length spin lock pulse. For the two compounds, the width of the π/2 pulse for ^1^H was 3.5 μs and the width of the π/2 pulse for ^13^C was 3.96–4.3 μs. The radiofrequency power of the spin-lock pulses was 71.42 kHz for ^1^H and 62.50 kHz for ^13^C. An almost constant temperature (error ± 0.5 K) was maintained even when the rate of flow of nitrogen gas and the heater current were adjusted.

## Experimental results

### X-ray results

**Figure 2:**
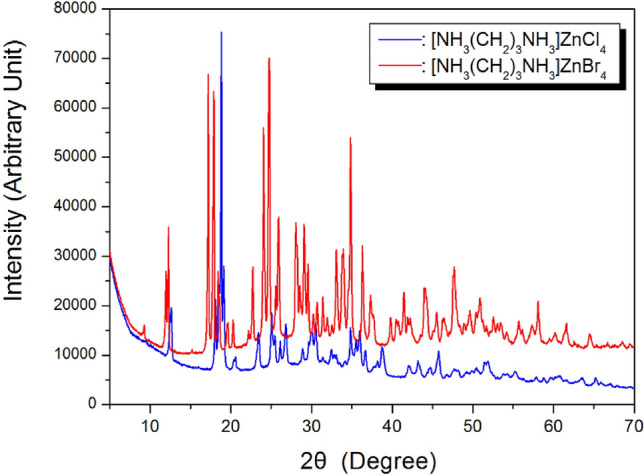
X-ray diffraction patterns of the [NH_3_(CH_2_)_3_NH_3_]ZnCl_4_ and [NH_3_(CH_2_)_3_NH_3_]ZnBr_4_ at 300 K.

The X-ray powder diffraction patterns of the [NH_3_(CH_2_)_3_NH_3_]Zn*X*_4_ (*X* = Cl and Br) crystals at 298 K are displayed in Fig. 2. And, the lattice constants for [NH_3_(CH_2_)_3_NH_3_]ZnCl_4_ were determined to be *a* = 10.670 Å, *b* = 10.576 Å, *c* = 10.755 Å, and β = 118.477°, and those for [NH_3_(CH_2_)_3_NH_3_]ZnBr_4_ were determined to be *a* = 11.146 Å, *b* = 10.995 Å, *c* = 11.188 Å, and β = 117.215°. These results are consistent with those reported previously^26,27^.

### Thermal properties

**Figure 3:**
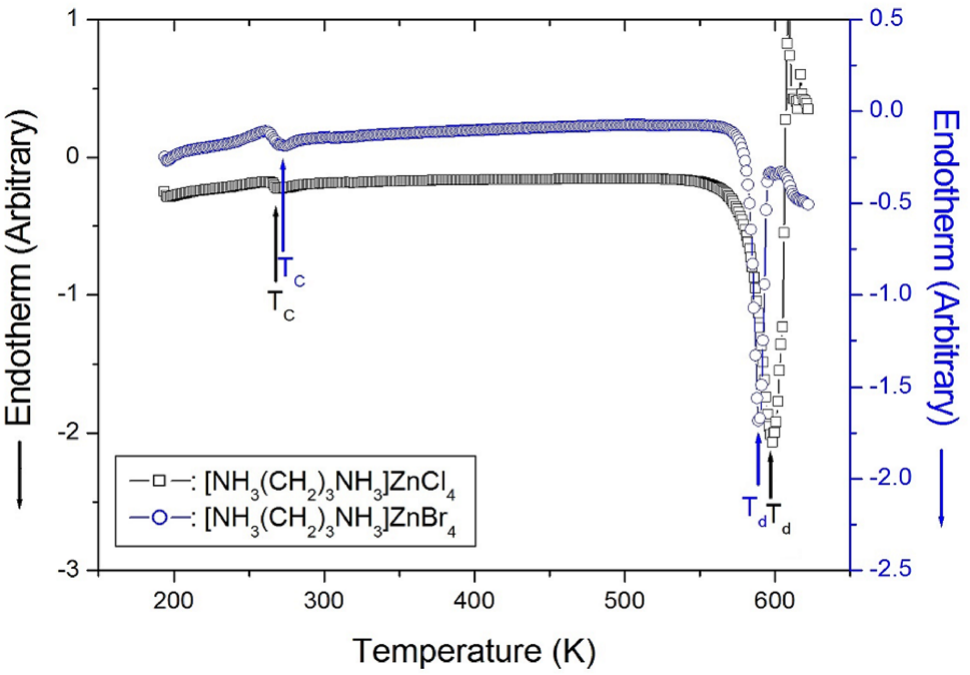
Differential scanning calorimetry (DSC) thermogram of [NH_3_(CH_2_)_3_NH_3_]ZnCl_4_ and [NH_3_(CH_2_)_3_NH_3_] ZnBr_4_.

**Figure 4:**
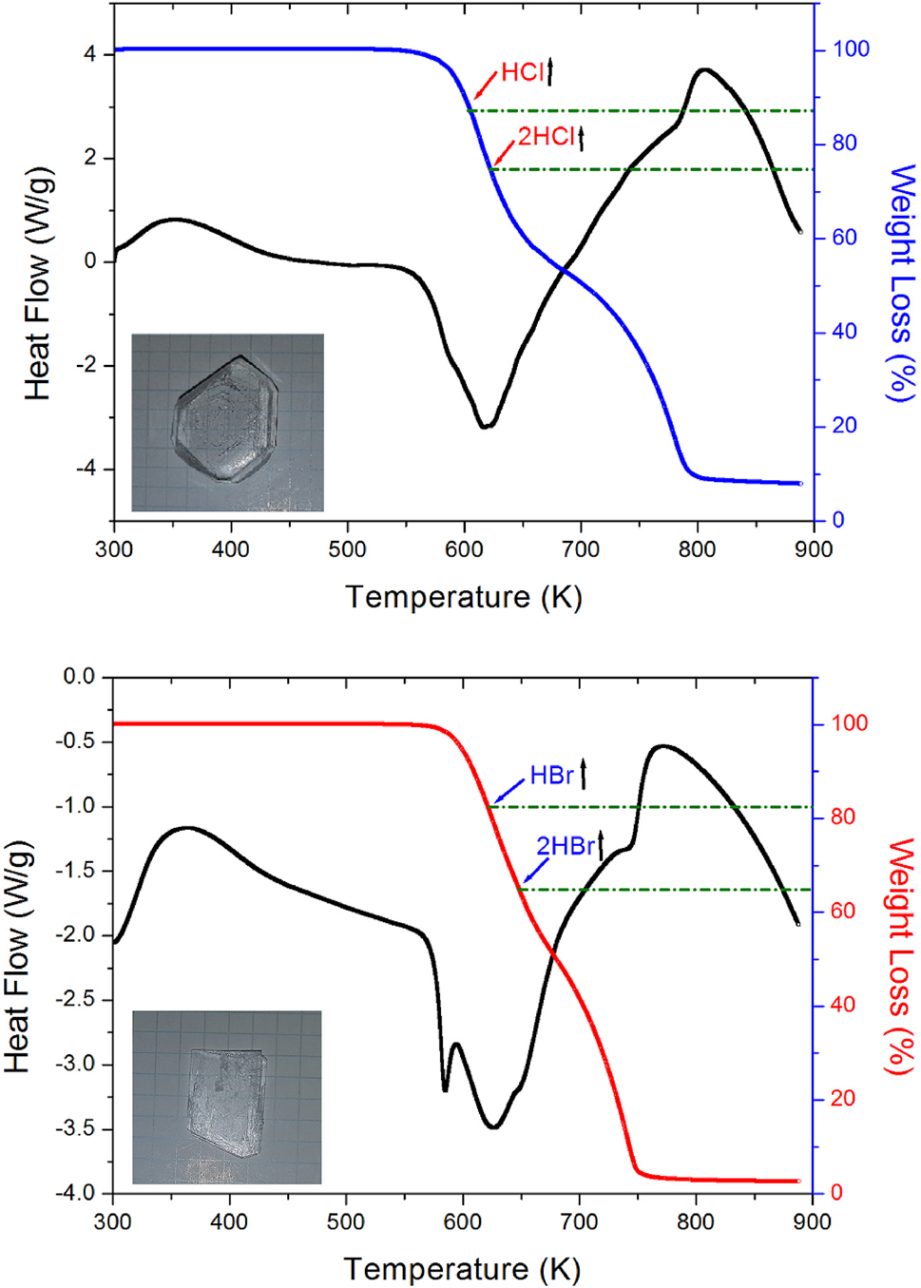
(**a**) Thermogravimetric analysis (TGA) and differential thermal analysis (DTA) of [NH_3_(CH_2_)_3_NH_3_]ZnCl_4_. (**b**). Thermogravimetric analysis (TGA) and differential thermal analysis (DTA) of [NH_3_(CH_2_)_3_NH_3_]ZnBr_4_.

Two endothermic peaks at 268 and 597 K were observed in the DSC curves of [NH_3_(CH_2_)_3_NH_3_]ZnCl_4_, as shown in Fig. 3. The DSC curves of [NH_3_(CH_2_)_3_NH_3_]ZnBr_4_ revealed the presence of two endothermic peaks at 272 and 589 K, as shown in Fig. 3. The results obtained from the DSC experiments revealed that the peaks that appeared at ~ 590 K were significantly larger than the other peaks. An additional TGA and differential thermal analysis (DTA) experiments were performed to determine whether these endothermic peaks are related to the structural phase transitions or melting. The results of the TGA and DTA experiments conducted with the two crystals are presented in Fig. 4a,b. The onset of thermal decomposition temperature (= T_d_) was observed at temperatures > 550 K. This was characterized by a loss in the weight of the compounds. It was observed that [NH_3_(CH_2_)_3_NH_3_]ZnCl_4_ (Mw = 283.34 mg) and [NH_3_(CH_2_)_3_NH_3_]ZnBr_4_ (Mw = 461.15 mg) started losing weight at higher temperatures. The amounts of solid residues obtained were calculated from the molecular weights of the compounds and balanced chemical reactions. The amounts were estimated from Eqs. (1), (2), (3), and (4)^28^.

### ***[NH***_***3***_***(CH***_***2***_***)***_***3***_***NH***_***3***_***]ZnCl***_***4***_

$$\left[ {{\text{NH}}_{3} \left( {{\text{CH}}_{2} } \right)_{3} {\text{NH}}_{3} } \right]{\text{ZnCl}}_{4} \to \left[ {{\text{NH}}_{2} \left( {{\text{CH}}_{2} } \right)_{3} {\text{NH}}_{2} \cdot2{\text{HCl}}} \right]{\text{ZnCl}}_{2} \to \left[ {{\text{NH}}_{2} \left( {{\text{CH}}_{2} } \right)_{3} {\text{NH}}_{2} \cdot{\text{HCl}}} \right]{\text{ZnCl}}_{2} \left( {\text{s}} \right) + {\text{HCl}}\left( {\text{g}} \right).$$

The amount of residue obtained is determined as follows:

1 $$\left[ {{\text{NH}}_{{2}} \left( {{\text{CH}}_{{2}} } \right)_{{3}} {\text{NH}}_{{2}} \cdot{\text{HCl}}} \right]{\text{ZnCl}}_{{2}} \left( {\text{s}} \right){/}\left[ {{\text{NH}}_{{2}} \left( {{\text{CH}}_{{2}} } \right)_{{3}} {\text{NH}}_{{2}} } \right]{\text{ZnCl}}_{{4}} = 87.13\% ,$$

$$\left[ {{\text{NH}}_{{3}} \left( {{\text{CH}}_{{2}} } \right)_{{3}} {\text{NH}}_{{3}} } \right]{\text{ZnCl}}_{{4}} \to \left[ {{\text{NH}}_{{2}} \left( {{\text{CH}}_{{2}} } \right)_{{3}} {\text{NH}}_{{2}} \cdot{\text{2HCl}}} \right]{\text{ZnCl}}_{{2}} \to \left[ {{\text{NH}}_{{2}} \left( {{\text{CH}}_{{2}} } \right)_{{3}} {\text{NH}}_{{2}} } \right]{\text{ZnCl}}_{{2}} \left( {\text{s}} \right) + 2{\text{HCl }}\left( {\text{g}} \right){.}$$

The amount of residue obtained is determined as follows:

2 $$\left[ {{\text{NH}}_{{2}} \left( {{\text{CH}}_{{2}} } \right)_{{3}} {\text{NH}}_{{2}} } \right]{\text{ZnCl}}_{{2}} \left( {\text{s}} \right)/\left[ {{\text{NH}}_{{2}} \left( {{\text{CH}}_{{2}} } \right)_{{3}} {\text{NH}}_{{2}} } \right]{\text{ZnCl}}_{4} = 74.26\% .$$

### ***[NH***_***3***_***(CH***_***2***_***)***_***3***_***NH***_***3***_***]ZnBr***_***4***_

$$\left[ {{\text{NH}}_{3} \left( {{\text{CH}}_{2} } \right)_{3} {\text{NH}}_{3} } \right]{\text{ZnBr}}_{4} \to \left[ {{\text{NH}}_{2} \left( {{\text{CH}}_{2} } \right)_{3} {\text{NH}}_{2} \cdot2{\text{HBr}}} \right]{\text{ZnBr}}_{2} \to \left[ {{\text{NH}}_{{\text{2}}} \left( {{\text{CH}}_{{\text{2}}} } \right)_{3} {\text{NH}}_{2} \cdot{\text{HBr}}} \right]{\text{ZnBr}}_{2} \left( {\text{s}} \right) + {\text{HBr}}\left( {\text{g}} \right).$$

The amount of residue obtained is determined as follows:

3 $$\left[ {{\text{NH}}_{{2}} \left( {{\text{CH}}_{{2}} } \right)_{{3}} {\text{NH}}_{{2}} \cdot{\text{HBr}}} \right]{\text{ZnBr}}_{2} \left( {\text{s}} \right)/\left[ {{\text{NH}}_{2} \left( {{\text{CH}}_{2} } \right)_{3} {\text{NH}}_{2} } \right]{\text{ZnBr}}_{4} = 82.45\% ,$$

$$\left[ {{\text{NH}}_{{\text{3}}} \left( {{\text{CH}}_{{\text{2}}} } \right)_{{\text{3}}} {\text{NH}}_{3} } \right]{\text{ZnBr}}_{4} \to \left[ {{\text{NH}}_{2} \left( {{\text{CH}}_{2} } \right)_{3} {\text{NH}}_{2} \cdot2{\text{HBr}}} \right]{\text{ZnBr}}_{2} \to \left[ {{\text{NH}}_{{\text{2}}} \left( {{\text{CH}}_{{\text{2}}} } \right)_{{\text{3}}} {\text{NH}}_{{\text{2}}} } \right]{\text{ZnBr}}_{{\text{2}}} \left( {\text{s}} \right) + 2{\text{HBr}}\;\left( {\text{g}} \right).$$

The amount of residue obtained is determined as follows:

4 $$\left[ {{\text{NH}}_{{2}} \left( {{\text{CH}}_{{2}} } \right)_{{3}} {\text{NH}}_{{2}} } \right]{\text{ZnBr}}_{{2}} \left( {\text{s}} \right){/}\left[ {{\text{NH}}_{{2}} \left( {{\text{CH}}_{{2}} } \right)_{{3}} {\text{NH}}_{{2}} } \right]{\text{ZnBr}}_{{4}} = {64}{\text{.91}}\% {.}$$

[NH_3_(CH_2_)_3_NH_3_]ZnCl_4_ was found to lose approximately 13% and 26% of its weight when the temperature was approximately 606 and 621 K, respectively. [NH_3_(CH_2_)_3_NH_3_]ZnBr_4_ was found to lose approximately 17% and 35% of its weight when the temperature was approximately 622 and 649 K, respectively. The weight loss can be potentially attributed to the decomposition of H*X* and 2H*X* (*X* = Cl and Br) moieties, respectively, as shown in Fig. 4a,b. Based on the DSC and TGA results, the phase transition temperatures for [NH_3_(CH_2_)_3_NH_3_]ZnCl_4_ and [NH_3_(CH_2_)_3_NH_3_]ZnBr_4_ were 268 K and 272 K, respectively. In addition, it was found that the endotherm peaks of 597 K for [NH_3_(CH_2_)_3_NH_3_]ZnCl_4_ and 589 K for [NH_3_(CH_2_)_3_NH_3_]ZnBr_4_ were related to T_d_.

### MAS ^1^H NMR results

**Figure 5:**
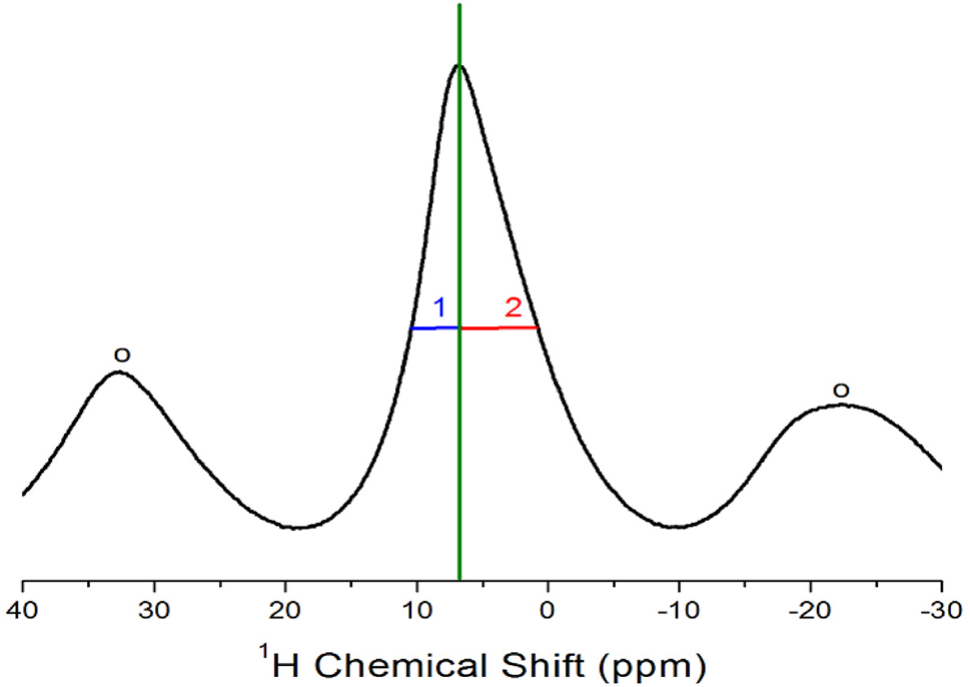
MAS ^1^H NMR line shapes of [NH_3_(CH_2_)_3_NH_3_]ZnBr_4_ at 300 K.

The temperature-induced ^1^H NMR chemical shifts for [NH_3_(CH_2_)_3_NH_3_]ZnCl_4_ and [NH_3_(CH_2_)_3_NH_3_]ZnBr_4_ crystals were recorded through MAS NMR spectroscopy. A single resonance signal was observed in each of the NMR spectra of both the compounds. The ^1^H NMR spectrum of [NH_3_(CH_2_)_3_NH_3_]ZnBr_4_ recorded at 300 K is shown in Fig. 5. The observed resonance signal was asymmetric. The line width represented as symbol 1 and that represented as symbol 2 at the full-width at half-maximum (FWHM) value are not the same as those at 3.54 and 6.11 ppm for 1 and 2, respectively. This asymmetric signal is attributed to the overlapping lines of the two ^1^H for CH_2_ and NH_3_ present in the [NH_3_(CH_2_)_3_NH_3_] cations. The spinning sidebands were marked with open circles. At 300 K, the ^1^H line width of the spectrum recorded for [NH_3_(CH_2_)_3_NH_3_]ZnCl_4_ was 10.57 ppm, whereas that of the spectrum recorded for [NH_3_(CH_2_)_3_NH_3_]ZnBr_4_ was 9.65 ppm. The ^1^H NMR chemical shifts for two ^1^H in the [NH_3_(CH_2_)_3_NH_3_] cations are temperature independent. The chemical shift values remained practically constant over a wide range of temperature, indicating that the structural environment of the protons present in the [NH_3_(CH_2_)_3_NH_3_] cation does not change when the temperature is increased.

**Figure 6:**
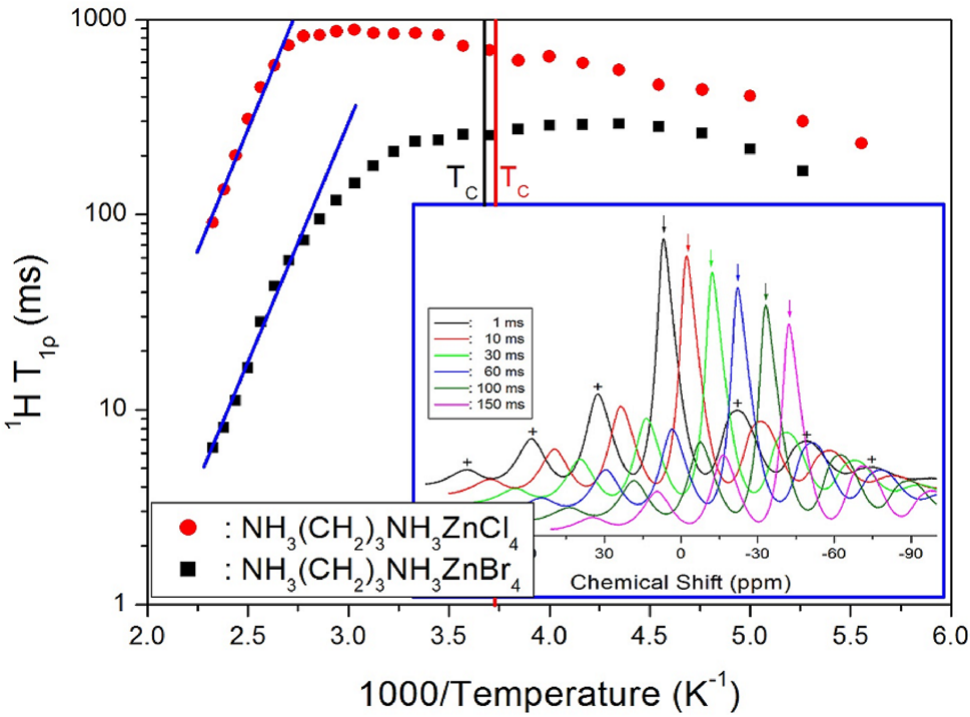
MAS ^1^H NMR spin–lattice relaxation times (T_1ρ_) of [NH_3_(CH_2_)_3_NH_3_]ZnCl_4_ and [NH_3_(CH_2_)_3_NH_3_]ZnBr_4_ as a function of inverse temperature (Inset: recovery curves for delay times of MAS ^1^H NMR spectrum in [NH_3_(CH_2_)_3_NH_3_]ZnCl_4_ at 300 K).

The ^1^H T_1ρ_ was measured at each temperature by applying a spin lock of variable duration, *t*. The relationship between the intensities of the NMR signals and the duration of the spin lock is as follows^29–31^:

5 $${\text{I}}\left( t \right) = {\text{I}}\left( 0 \right){\text{exp}}( - t/{\text{T}}_{1\rho } ),$$

where I(*t*) and I(0) are the signal intensities at times *t* and *t* = 0, respectively. The ^1^H NMR signals were plotted as a function of the delay times at each temperature. The ^1^H NMR signals for [NH_3_(CH_2_)_3_NH_3_]ZnCl_4_ at 300 K were recorded by varying the delay times (in the range of 1–150 ms, Fig. 6). The decay curves were fit to an exponential function, as shown in Eq. (5). The T_1ρ_ values of the protons in [NH_3_(CH_2_)_3_NH_3_]ZnCl_4_ and [NH_3_(CH_2_)_3_NH_3_]ZnBr_4_ were obtained as a function of inverse temperature, as shown in Fig. 6. The T_1ρ_ values of the atoms in the two compounds did not change near T_C_ (268 K for [NH_3_(CH_2_)_3_NH_3_]ZnCl_4_ and 272 K for [NH_3_(CH_2_)_3_NH_3_]ZnBr_4_). The T_1ρ_ values initially increased and then decreased when the temperature was increased. In addition, the trend of changes in the ^1^H T_1ρ_ values of [NH_3_(CH_2_)_3_NH_3_]ZnBr_4_ and [NH_3_(CH_2_)_3_NH_3_]ZnCl_4_ with temperature was similar. The T_1ρ_ values of the protons in [NH_3_(CH_2_)_3_NH_3_] in both the crystals were 10–1000 ms. The ^1^H T_1ρ_ values of [NH_3_(CH_2_)_3_NH_3_]ZnCl_4_ decreased abruptly at 360 K and that of [NH_3_(CH_2_)_3_NH_3_]ZnBr_4_ decreased abruptly at 300 K (as represented by lines). The T_1ρ_ value of the protons in [NH_3_(CH_2_)_3_NH_3_]ZnCl_4_ was higher than that in [NH_3_(CH_2_)_3_NH_3_]ZnBr_4_ at all studied temperatures. For both the compounds, lower T_1ρ_ values were recorded at higher temperatures. On the slow side of T_1ρ_, a decrease in T_1ρ_ results in smaller valued of the correlation time τ_C_. The T_1ρ_ values for Arrhenius-type random motions with τ_C_ are described in terms of slow motions. When τ_C_ <  < ω_1_, T_1ρ_ ∝ τ_C_ = τ_0_ exp(-E_a_/k_B_T), where ω_1_ denotes the spin-lock frequency and E_a_ represents the activation energy. The decrease in T_1ρ_ values with temperature indicates an increase in proton mobility at higher temperatures.

### MAS ^13^C NMR results

**Figure 7:**
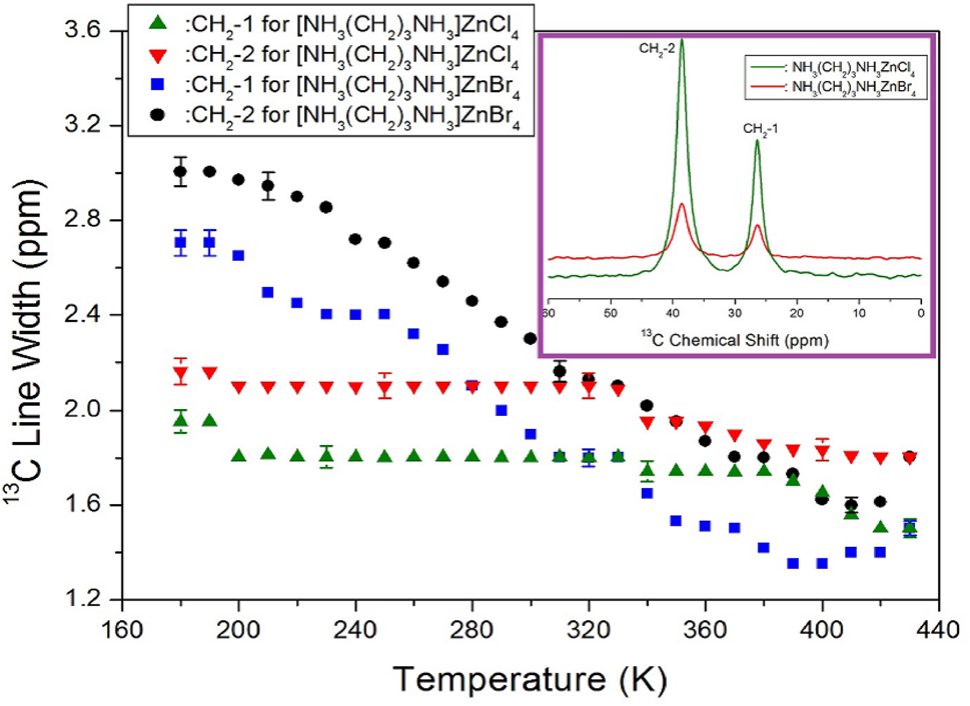
Line widths of ^13^C for CH_2_-1 and CH_2_-2 in [NH_3_(CH_2_)_3_NH_3_]ZnCl_4_ and [NH_3_(CH_2_)_3_NH_3_]ZnBr_4_ as a function of temperature (Inset: MAS ^13^C NMR spectra of [NH_3_(CH_2_)_3_NH_3_]ZnCl_4_ and [NH_3_(CH_2_)_3_NH_3_] ZnBr_4_ at 300 K).

The MAS ^13^C NMR chemical shifts in [NH_3_(CH_2_)_3_NH_3_]ZnCl_4_ and [NH_3_(CH_2_)_3_NH_3_]ZnBr_4_ were measured according to the change in temperature. Two resonance signals were observed in the MAS ^13^C NMR spectra of the compounds. The chemical shift values of the other carbon atoms were determined relative to the TMS signal. Here, the CH_2_ group sandwiched between two other CH_2_ groups is labeled as CH_2_-1. The CH_2_ group close to NH_3_ is labeled CH_2_-2. At 300 K, the signals corresponding to the CH_2_-1 and CH_2_-2 carbon atoms in [NH_3_(CH_2_)_3_NH_3_]ZnCl_4_ appear at δ = 26.41 and δ = 38.53 ppm, respectively. The chemical shift values of the carbon atoms in [NH_3_(CH_2_)_3_NH_3_]ZnBr_4_ are similar to the chemical shift values of the carbon atoms in [NH_3_(CH_2_)_3_NH_3_]ZnCl_4_ (Fig. 7, inset). It was observed that for both the crystals, the ^13^C chemical shift values remained almost constant with changes in temperature (Supplementary Fig. 1).

The change in FWHM for ^13^C NMR spectra with temperature for both crystals is shown in Fig. 7. In the case of [NH_3_(CH_2_)_3_NH_3_]ZnCl_4_, the ^13^C line widths (CH_2_-1 and CH_2_-2) decreased monotonically with an increase in temperature. Significant anomaly due to phase transition was not observed. The ^13^C line widths in [NH_3_(CH_2_)_3_NH_3_]ZnBr_4_ changed with the increase in temperature. A transformation from a Gaussian to Lorentzian shape was observed. The line width reduced because of internal molecular motion. The line width of CH_2_-1 was lower than that of CH_2_-2. The decrease in line width with an increase in temperature can be attributed to the internal molecular motion.

**Figure 8:**
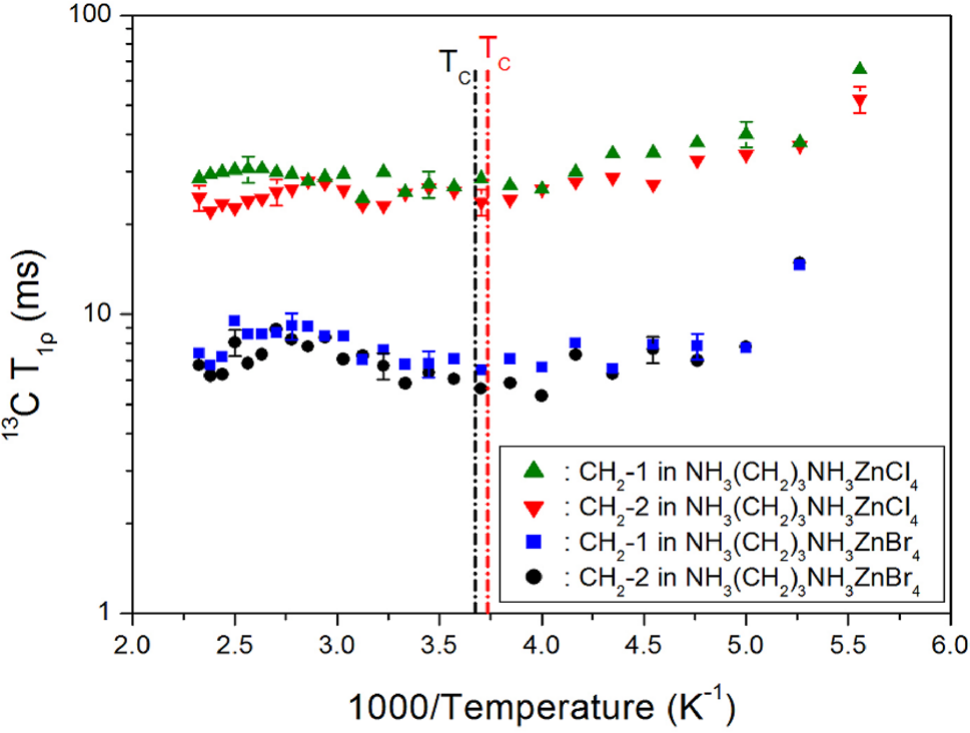
T_1ρ_ for CH_2_-1 and CH_2_-2 of [NH_3_(CH_2_)_3_NH_3_]ZnCl_4_ and [NH_3_(CH_2_)_3_NH_3_]ZnBr_4_ as a function of inverse temperature.

The intensities of the ^13^C NMR signals were determined by varying the delay times at each temperature. The ^13^C NMR signals (CH_2_-1 and CH_2_-2) recorded for [NH_3_(CH_2_)_3_NH_3_]ZnCl_4_ and [NH_3_(CH_2_)_3_NH_3_]ZnBr_4_ were plotted as a function of delay time*.* The decay curves for CH_2_-1 and CH_2_-2 were fitted to an exponential equation of Eq. (5). From the slopes of the recovery traces, the ^13^C T_1ρ_ values were obtained (CH_2_-1 and CH_2_-2) as a function of inverse temperature, as shown in Fig. 8. The ^13^C T_1ρ_ values of [NH_3_(CH_2_)_3_NH_3_]ZnCl_4_ were higher than those of [NH_3_(CH_2_)_3_NH_3_]ZnBr_4_. The ^13^C T_1ρ_ value of CH_2_-1 was slightly higher than the ^13^C T_1ρ_ value of CH_2_-2. The T_1ρ_ values of the atoms in both the compounds were temperature independent at > 200 K.

## Conclusion

The physical properties of organic–inorganic hybrid crystals [NH_3_(CH_2_)_3_NH_3_]Zn*X*_4_ (*X* = Cl, Br) were investigated through DSC, TGA, and NMR spectroscopy. The TGA results revealed that the [NH_3_(CH_2_)_3_NH_3_]Zn*X*_4_ (*X* = Cl, Br) complex exhibited good thermal stability. When the temperature was increased, the ^1^H and ^13^C chemical shifts remained almost constant. Although the T_C_ was obtained from the DSC experiments, in particular, changes in the chemical shift values were not observed at temperatures near T_C_. This indicated that the chemical environment surrounding the protons and the carbon atoms in the [NH_3_(CH_2_)_3_NH_3_] cation remained unaltered.

The structural dynamics were discussed in terms of the ^1^H T_1ρ_ and ^13^C T_1ρ_ values. The ^1^H T_1ρ_ values changed with the temperature, whereas the T_1ρ_ value of the carbon atom located in the middle of the N–C–C–C–N chain did not change significantly with the temperature. The ^1^H T_1ρ_ and ^13^C T_1ρ_ values of the atoms in [NH_3_(CH_2_)_3_NH_3_]ZnCl_4_ were higher than those in [NH_3_(CH_2_)_3_NH_3_]ZnBr_4_ (Table 1). The reason why ^13^C T_1ρ_ values in [NH_3_(CH_2_)_3_NH_3_]ZnCl_4_ are longer than ^13^C T_1ρ_ values in [NH_3_(CH_2_)_3_NH_3_]ZnBr_4_ are as follows. The ^13^C T_1ρ_ is most likely driven by ^1^H-^13^C interactions, and hence, the H-C bond lengths are important.

**Table 1:** The lattice constants (Å), phase transition temperatures T_C_ (K), thermal decomposition temperatures T_d_ (K), and spin–lattice relaxation times T_1ρ_ (*ms*) in [NH_3_(CH_2_)_3_NH_3_]Zn*X*_4_ (*X* = Cl and Br) crystals at 300 K.

	[NH_3_(CH_2_)_3_NH_3_]ZnCl_4_	[NH_3_(CH_2_)_3_NH_3_]ZnBr_4_
Lattice constants	*a* = 10.670, *b* = 10.576, *c* = 10.755, *β* = 118.477	*a* = 11.146, *b* = 10.995, *c* = 11.188, *β* = 117.215°
T_C_	268	272
T_d_	597	589
^1^H T_1ρ_	849	234
^13^C T_1ρ_ for CH_2_-1	18.78	5.86
^13^C T_1ρ_ for CH_2_-2	21.05	6.80

However, the H-C bond lengths of the two materials are unlikely to be different, and also cannot be accurately measured from X-ray results. We assume that the differences in the dynamics between the materials contribute more to the difference in T_1ρ_. Although the structures and lattice constants of two crystals are similar, the differences between the local environments and structural dynamics obtained from the chemical shifts and T_1ρ_ values of the two compounds can be attributed to the different bond lengths and halogen atoms. Thus, the results of this study elucidate, the physical properties of [NH_3_(CH_2_)_3_NH_3_]Zn*X*_4_, which will expand the application scope of this organic–inorganic hybrid crystals.

## Supplementary Information

Supplementary Information
