# Supplementary material for: Physicochemical properties and structural dynamics of organic–inorganic hybrid [NH3(CH2)3NH3]ZnX4 (X = Cl and Br) crystals
Source: Sci Rep. 2021 Apr 16;11:8408. doi: 10.1038/s41598-021-87940-2 (PMC8052450; doi:10.1038/s41598-021-87940-2)
Supplement: Supplementary file 1 — Supplementary Information [file 41598_2021_87940_MOESM1_ESM.docx]

**Physicochemical properties and structural dynamics of organic–inorganic hybrid perovskite [NH_3_(CH_2_)_3_NH_3_]Zn*X*_4_ (*X*=Cl and Br) crystals**

Ae Ran Lim^1, 2, *^, Sun Ha Kim^3, 4^, Yong Lak Joo^5^

^1^Department of Carbon Convergence Engineering, Jeonju University, Jeonju 55069, Korea.

^2^Department of Science Education, Jeonju University, Jeonju 55069, Korea. email: [arlim@jj.ac.kr](mailto:arlim@jj.ac.kr)

^3^Korea Basic Science Institute, Seoul Western Center, Seoul 03759, Korea.

^4^Department of Chemistry, Kyungpook National University, Daegu 41566, Korea.

^5^Robert Fredrick Smith School of Chemical and Biomolecular Engineering, Cornell University, Ithaca, NY 14853, USA.


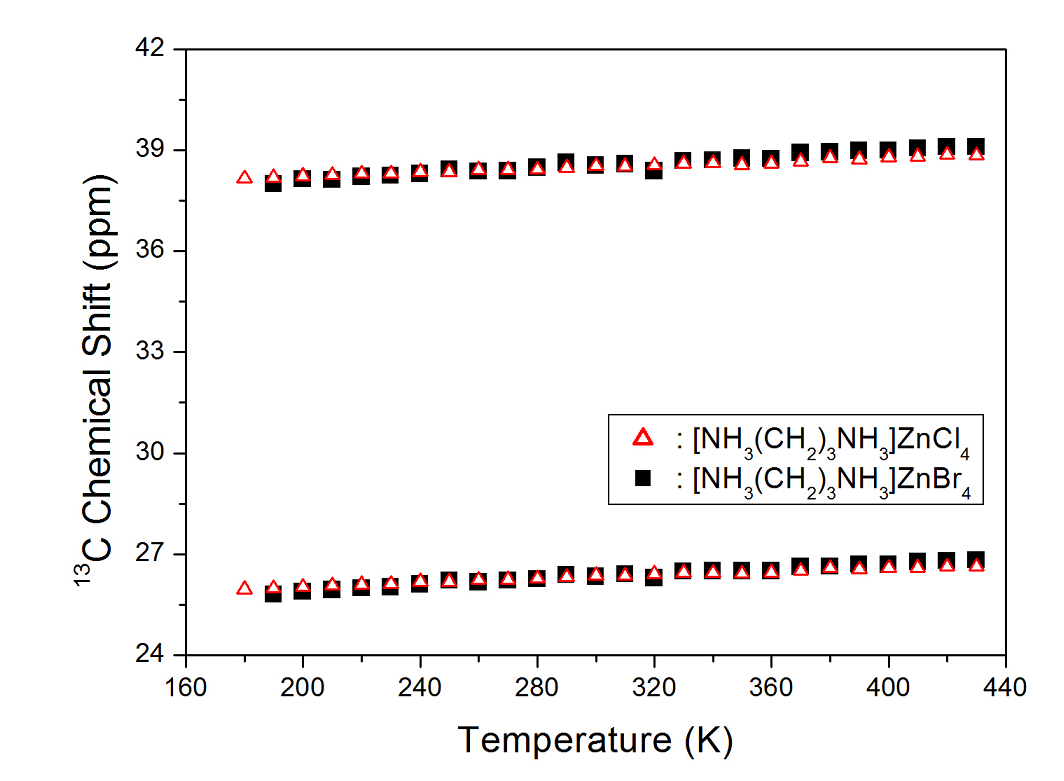


Supplementary Figure 1. Chemical shifts of [NH_3_(CH_2_)_3_NH_3_]Zn*X*_4_ (*X*=Cl and Br) as a function of temperature.
